# Pathology

**Published:** 1824-09-01

**Authors:** 


### PATHOLOGY.

1. *Paralysis on the same side as the Cerebral Injury**. There is no law in pathology more general than that the paralysis should be situated in the side opposite to that where the pressure is exercised on the brain; and yet there are exceptions even to this rule. Several modern pathologists have doubted these exceptions; but, for our own parts, we do not doubt them, because we have seen two unequivocal examples in our own practice. Lieutaud relates one instance, but not circumstantially. We shall begin with the case of the celebrated Malpighi, as recorded by Baglivi.

*Case* 1. Marcellus Malpighi, aged 70, had been subject, for some years, to bilious vomitings, which being suppressed, he had acidity of the stomach, palpitations of the heart, and both renal and vesical calculi. On the 25th July, 1694, he was struck with apoplexy, followed by paralysis of the whole of the right side. Bleeding, and other evacuations were prescribed by Baglivi, and after forty days the patient recovered of the apoplexy, and even of the paralysis; the memory, however, and some other of the intellectual functions, remaining seriously deteriorated. On the 29th of November of the same year, he was again struck with apoplexy, preceded by much vertiginous affection, and this attack proved fatal in a few hours. On dissection, an enormous

* M. Bayle. Revue Med. Janvier, 1824.

quantity of blood was found in the *right* ventricle of the brain, and only a little serum in the *left*.

This case certainly goes for nothing in support of the question here discussed, though it was brought forward for this purpose, by Baglivi himself.

It is abundantly evident that the quantity of blood found effused, on dissection, was the immediate cause of death, and could only have been extravasated in the *last* attack of apoplexy. The cure of the hemiplegia in forty days after the first attack, is a sufficient proof that there was only congestion of vessels, not extravasation of blood, during that attack. The matter was put beyond a doubt, by the absence of any clot or cyst in the brain after death.

*Case* 2. A porter received a contusion on the *left temple*, with slight fracture of the bone. He was trephined. Nevertheless he was affected with paralysis of the *right* arm. He died in three days, and, on dissection, blood was found extravasated on the *right* hemisphere of the brain, and none on the *left*.—*Smetius*.

This case, though not sufficiently detailed, proves that the paralysis may be on the same side as the sanguineous effusion. But then, it may be queried whether the paralysis of the *right* arm was owing to the effusion of blood on the *right* hemisphere of the brain, or to the injury done to the *left* hemisphere by the contusion and fracture? We should be inclined to take the last side of the question.

*Case* 3. A child, two years of age, was seized with a lethargy or stupor, but not in a very severe degree. He recovered from this state, and again relapsed into a still more profound lethargy. In this last attack, the *right* side was paralysed and insensible. He died on the fourth day. On dissection, Forestus (who relates the case) found the *right* side, both of the cerebrum and cerebellum, entirely disorganized, and affected not only with abscess, but with sphacelus. On the *left* side all was sound. Although the symptoms are detailed in an unsatisfactory manner, and we know of no such thing as *sphacelus* of the brain, in the present day, yet it is undeniable that the paralysis was on the same side as the organic lesion in the brain, whatever was the real nature of that lesion.

*Case* 4. A man, upwards of seventy years of age, and who, for some years, had exhibited symptoms of cerebral disorder, was suddenly seized with paralysis and loss of sense in the whole *right* side of the body. He died in a few hours. On dissection, blood was found extravasated in the ventricles. A large ulceration, whence the blood had proceeded, was found to occupy the place of the *right* thalamus optici.—*Valsalva*.

Some doubt is thrown on the pathology of this case, in consequence of the extravasated blood having penetrated into *both* ventricles; for in this case *pressure* must have been made on both sides of the brain. It ought, however, according to the general doctrine, to have been greater in the *right*, than in the left ventricle, and, consequently, the paralysis ought to have been in the *left* side of the body, rather than in the right.

*Case* 5. A woman, forty-seven years of age, was seized with apoplexy, in the month of November, 1680, and the *right* side of the body completely deprived of sense and motion. Brunner (who relates the case) prescribed blood-letting, strong counter-irritation, and ultimately purgatives. She gradually recovered the use of the paralysed side, though a weakness remained in the parts. After this attack, her disposition became quite altered, and she addicted herself to drinking strong liquors, which brought on her the reproaches of her husband, and sometimes corrections of a more tangible and material form. She was subject to vertiginous affections and cephalalgia. Eight years afterwards, she was again seized with a severe attack of apoplexy, and sudden loss of sense. She died in a few hours. On dissection, the cause of the former attack of apoplexy, viz. the remains of a clot of blood, was found in the *right* hemisphere of the brain, and the cerebral substance surrounding the clot, of a yellow colour, and of a denser structure than elsewhere. Two other smaller caverns, or remains of extravasation, were also discovered in the same hemisphere. The right ventricle was full of blood, and some had penetrated through the septum lucidum into the left ventricle. A recent clot, or coagulum of extravasated blood, was found in the right hemisphere—the cause, of course, of the last or fatal attack of apoplexy. . The *left* side of the brain was sound.

This case is more complete in its details (many of which we have omitted) than any of the preceding. We think it incontestibly proves that paralysis may sometimes be on the same side as the cerebral lesion, that occasioned it.

*Case* 6. An old woman was seized with apoplexy, accompanied and followed by paralysis (loss of motion, but not of sense) in the upper and lower extremities of the *right* side. Three months afterwards, she fell into a state of languor and debility, of which she died in an hospital in a few days. The *left* hemisphere of the brain was perfectly sound. Morgagni (the relator of the case) discovered a remarkable disorganization (a softening) of the brain in the *right* hemisphere, which was evidently the cause of the apoplexy and paralysis that took place three months before the patient's death.

*Case* 7. This case was also observed by Morgagni. A female peasant, twenty-four years of age, six months gone in pregnancy, was seized with apoplexy, and also paralysis of the *right* side of the body. Abortion quickly took place, and she expired half an hour after this event. Morgagni demonstrated the brain of this woman, in his public lectures of 1724. He found a serous effusion under the arachnoid membrane; but what was of more importance, a very large clot of blood in the substance of the *right* hemisphere of the brain. No cognizable derangement could be traced in the *left* side of the encephalon.

This case was so very conclusive, and militated so strongly against the doctrine maintained by Morgagni himself, that, distrusting his own observation, he asked all the students who attended the case, whether they were certain that the paralysis was on the *right* side of the body. But they, one and all, replied in the affirmative.

*Case* 8. A soldier, thirty-three years of age, became affected with temporary insanity. In the course of a month, reason was restored; but he continued weak, and this weakness was followed by a series of epileptic attacks of an irregular kind, which left much intellectual as well as corporeal debility behind. In the course of eight months, the patient lost entirely the power of speech, and three weeks after this event he was seized with apoplexy, and general loss of motion and feeling. On the second day, the *left* arm was paralytic, the sensibility remaining. On the fourth day he died. On dissection, there was found an intimate adhesion of the tunica arachnoidea and dura mater, on the anterior portion of the *left* hemisphere, both membranes being thickened and inflamed, the latter covered with membraniform exudations. Beneath this part, the brain itself was softened and disorganized, resembling so much bouillie, its central portion being almost fluid. The whole of the *right* side of the brain was sound.—*Morgagni.*

This case appears fully as conclusive as any that preceded. Dr. Bayle does not adduce any modern instances, which he might have done. We have seen two or three unequivocal examples of paralysis and cerebral injury on the same side, one of which we published in the first volume of the Transactions of the Associated Apothecaries.

The question now recurs, why do we find these exceptions to a law so universal in pathological physiology? Areteus first annunciated the law of opposite paralysis and injury, and after his time the rule was considered as absolute, while they gave the most erroneous and even ridiculous explanations of the law. Mistichelli appears to have been the first who offered a natural and true solution of the problem, by discovering the interlacing; or, as it may be termed, decussation of the nervous fasciculi, composing the medulla oblongata. After this decussation was ascertained, the problem respecting paralysis on the opposite side was not only solved, but the rule was now considered more absolute than ever, and exceptions impossible. Winslow, Santorini, and Morgagni, confirmed the discovery of Mistichelli; but yet the discovery was almost forgotten, till Dr. Gall lately demonstrated the decussation or twisting of the cerebral fibres in the medulla oblongata, since which he has been generally considered the discoverer. Our readers are aware that M. Serres, and some other continental writers have recently denied the possibility of the paralysis being on the same side as the injury—in the face of facts, such as those we have just cited. But accurate anatomical investigation has shewn that we should not be too precipitate in coming either to conclusions or exclusions, on this point; since, although there is a general twisting of the nervous filaments in the medulla oblongata, there is *not a total change of sides.* Some fibres are found *not* to decussate, but to continue from the brain to the spinal marrow—or, if it must be so, from the spinal marrow to the brain, on the same side. This fact, so amply proved and demonstrated by Gall, offers the only rational solution which we yet possess of the exceptions to the general rule in question. It is curious that even this piece of minute anatomy did not escape the penetrating eye of the illustrious Morgagni. At the conclusion of case 7, above cited, he observes—"how shall we account for the paralysis being on the same side as the cerebral lesion? Although there are a great many nervous filaments of the brain, which are continued into the medulla oblongata et spinalis, and from thence into the nerves, yet it is not certain that they *all* decussate. It is highly probable that there are parts in the hemispheres of the brain, from which cerebral filaments are continued along the *same side* to the spinal marrow and nerves—and it is also probable that these are the portions of the brain in which the lesions are situated, when the paralysis appears on the same side as the cerebral injury."—*Loco citato.*

At the same time, it must be confessed that the explanation here attempted is liable to some weighty objections, which cannot be completely solved in the present state of our knowledge. Thus, when we consider that there are but a very few cerebral filaments which do *not* change sides, or decussate in the medulla oblongata, and, consequently, that there can be but a few points in the encephalon from which these fibres or filaments originate, it seems unaccountable how large disorganizations or dilacerations of the brain should only involve these minute origins, leaving the other, or decussating portions untouched. But, although the causes are concealed from our view, the facts are not less certain—that paralysis and cerebral lesion may be occasionally on the same side.

---

2. *Melæna.* Dr. Martland (*Ed. Journal, No.* 2) has given us a case of melæna, with the appearances on dissection. The patient was forty-nine years of age, addicted to dram-drinking. On the 16th July, 1823, when Dr. M. was called to him, his alvine evacuations were of the colour and consistence of liquid pitch, remarkably fetid, but in small quantity. He had no pain in the abdomen, but felt an oppressive weight about the præcordia. The strength was impaired, countenance pallid and very anxious—pulse 110, and weak—skin hot and dry—urine high-coloured and scanty—vertigo. A saline cathartic, and afterwards powders composed of nitre and the compound powder of ipecacuan. Most of the symptoms were relieved by this plan, but the stools continued the same. He then took a mixture composed of nitre, sulphate of magnesia, and snlphuric acid. The stools now became nearly natural. Dropsical effusion in the abdomen and lower extremeties supervened. Mercury, elaterium, digitalis, opium, nitre, juniper-top infusion. By these remedies, the dropsy was removed. In October, the melæna returned in an aggravated form. He vomited up half a pint of black grumous blood, became comatose, and died.

*Dissection, 48 hours after death.* Black grumous blood had issued from the mouth since death, and pressure on the abdomen caused it to be forcibly discharged. Much fat in the omentum—the whole alimentary canal much distended with fetid gas—half a pint of black grumous blood in the stomach—but little of the same in the intestines—no proper fæces to be seen—peritoneal coat transparent, and shewing the villous coat throughout the whole canal—the latter coat was inflamed in every part, assuming a gangrenous hue, at the cardiac and pyloric extremeties. In the duodenum, the inflammation was trifling, but it increased gradually towards the ileum, where it seemed in the worst state of gangrene. No effusion between the coats of the intestines. From the termination of the ileum to the descending branch of the colon, were progressively exhibited different appearances, from the gangrenous inflammation to a blush. The colon was pretty healthy, but the villous coat of the rectum was gangrenous. The liver was of a pale brown colour, and smaller than usual, with a shrivelled fissured surface. It contained neither blood nor bile. There was no unusual appearance in any of the other viscera or parts.

The profession is indebted to Dr. Martland for this minute dissection of a disease comparatively rare in its occurrence, and little understood in its pathology.

---

3. *Organic Changes in the Bronchia.* The younger Andral, already favourably known to the profession by his pathological researches, has published a paper on the subject in question, through the medium of the *Archives Generales* for April last. The cases brought forward in illustration were treated in M. Lerminier's wards, in La Charite. They are, therefore, well authenticated.

It is well known that Laennec, in his valuable work on "Mediate Auscultation," drew the attention of the profession to this organic change in the air tubes, but the facts which he had then collected were scanty, and required confirmation by others. Andral avers, that his own personal experience has verified the proposition of Laennec—and that he has constantly found *dilatation* of the bronchia, when it existed, accompanied by chronic pulmonary catarrh—and that when the dilatation was considerable, it was announced by a resonance of the voice (resonnance de la voix) resembling pectoriloquism. In a less degree, the bronchial dilatation was still discoverable by characteristic signs—but in a very slight degree; it was not to be detected by any outward sign. Examples of the different shades are found in the cases detailed, of which we shall abbreviate the more important, for the benefit of the English reader.

*Case* 1. *Dilatation.* A porter, sixty-six years of age, entered La Charite in the beginning of January 1822, presenting all the symptoms of organic disease of the heart, as orthopnœa, puffiness of the face, anasarca, extended pulsation of the heart, &c. On applying the stethoscope between the right clavicle and the nipple of that side, a sound somewhat resembling pectoriloquism was heard, and in that part only.

The patient died suddenly a few days afterwards. On dissection, the lungs were found gorged with a serous fluid, and the bronchia of the right lobes, especially at the upper part, were greatly dilated as compared with those of the left side. In two or three places the mucous membrane, lining the bronchia, was ulcerated. There was hypertrophy of the ventricles, with dilatation of their cavities.

*Case* 2. A man, 62 years of age, was received into the hospital, in the month of April, 1822, who had been affected with cough and expectoration for five or six years. He had lately presented symptoms also of organic disease of the stomach. When he entered the hospital, he was in the advanced state of marasmus—frequent cough—much expectoration of a thick and yellow matter. No particular diagnosis could be got from the stethoscope. He sunk from the disease of the stomach. On dissection, the pulmonary parenchyma was healthy and crepitous throughout. In the middle lobe of the right side was found a portion of bronchium dilated to three times the size of that immediately above it, and which, in a healthy state, would have been proportionally smaller. There was ulceration of the pyloric orifice of the stomach.

This partial and limited dilatation did not appear to exercise any particular influence, and was inappreciable by any external phenomena. The case is chiefly valuable as shewing what a large quantity of purulent and thick expectoration can issue from the mucous membrane of the lungs, without any disease of the parenchymatous structure.

*Case* 3. A hair-dresser, forty-six years of age, was subject to catarrh with expectoration, for some years. Towards the end of 1821, he had an attack of hæmoptysis, for the first time. In February 1822, he expectorated large quantities of puriform and highly fetid matter. For eight days previous to his entrance into La Charite, he felt a sharp pain in the left side of the chest. He was received into the hospital in the end of March, having orthopnœa, and much anxiety of countenance. He expectorated, without difficulty, a thick yellow matter, mixed with considerable quantity of phlegm. He had so much pain in the left side of the chest, as to be unable to bear percussion of that part. The application of the stethoscope discovered evident pectoriloquism in the left side. M. Lerminier pronounced the patient to be affected with phthisis pulmonalis, of a slow progress. The nature of the expectoration, and the pectoriloquism indicated the existence of excavations in the lungs. There was also reason to suspect inflammation of the serous membranes in that side of the chest. The want of respiratory murmur in the left side of the thorax led to the suspicion that effusion had taken place there; or that the pain prevented the action of the respiratory muscles, and, consequently, the introduction of air into that lung, in the usual quantity. In the first days of April, the expectoration changed its character, being thinner, but still very fetid, and so copious as to fill three common spitting-pots in the twenty-four hours. During April and May the patient became progressively emaciated—the pain in the side continued, and prevented his lying on that side—expectoration still more fetid—cold chills in the evening—burning heats in the night—no morning perspirations—a circumstance that appeared strange, in a man presumptively labouring under tubercular consumption. In the mornings, and through the day, the pulse was but little accelerated. The patient lost all relish for food—yet the tongue was natural—never had vomiting or epigastric uneasiness. In May, a diarrhœa came on—then stopped, and re-appeared alternately. He sunk in the middle of June.

*Dissection.* Great marasmus. Several patches of false membrane were spread here and there over the left pleura pulmonalis—no adhesions between it and the pleura costalis—leftlung little crepitous—yet it swam in water. In the superior lobe of this lung there was a cavity the size of a wall-nut filled with matter resembling that expectorated. Into this cavity opened a bronchial tube that would admit a writing quill. This dilated tube and the cavity were lined with a similar membrane, which was reddened and thickened. It was now evident that what had been taken for a tubercular excavation was no other than a bronchial dilatation. Into the internal surface of this dilated portion several small openings were seen that led into other bronchia. In pursuing the various bronchial ramifications of the left lung generally, numerous dilatations, though on a smaller scale, were discovered—none of them, however, terminating in a cul de sac, as the above. The parenchymatous structure of the lung between these dilatations, was somewhat denser than natural, as if compressed by the dilated bronchia. The right lung, which was much more crepitous than the left, presented also some bronchial dilatations, but to a much less extent than on the other side. They were filled with a puriform liquid—and some of them were large enough to contain a hazel nut. There was no other disease in the chest. There were some small ulcerations in the mucous membrane of the stomach; and the internal surface of the transverse and descending colon was highly injected. These last were of recent occurrence, of course, and corresponded with the period of the bowel complaint.

In the above case it will be seen that even under the eyes of Laennec himself, a dilated bronchial tube caused the phenomenon of pectoriloquism, so indicative of tubercular excavations. In the second place, the case exhibits almost every symptom of tubercular phthisis (excepting only the morning perspirations) where the parenchymatous structure of the lungs was, we may say, perfectly sound. We also find that this kind of phthisis destroys life, with nearly the same certainty as the tubercular species, though there would be greater hope, in such cases, of a procrastinated fate. What could cause the fœtor of the expectoration?—It is curious that, in a case of Laennec's, where the same kind of disease was found after death, there was a similar fœtor of the breath.

*Case* 3. A man, 50 years of age, fell a victim to organic disease of the liver. He had long been affected with obstinate cough, and copious puriform expectoration. Auscultation was frequently tried at the hospital, and the mucous rattling (râle muqueux) was very evident in the left side of the chest, both before and behind. The resonance of the voice was not greater there than natural. Percussion elicited a clear sound. This man evinced no symptom of tuberculous degeneration in the lungs—the "râle muqueux" appearing to depend merely on accumulation of mucus in the bronchia. Dissection justified this opinion. The greater number of bronchial tubes in the left lung were red, on their internal surface, and filled with puriform mucus. There were several bronchial dilatations in this lung, filled also with similar matter.

*Stricture of the Bronchia.* This is a much more rare disease than dilatation. Like this last, it may occupy a single bronchial tube, partially or totally—or it may take place in several—nay, in all the bronchia of an entire lobe. As in dilatation, so in contraction, the cause may be chronic inflammation, ending in thickening of bronchial parietes. In some cases, however, it may be owing to mechanical compression of a bronchial tube from contiguous tumours. The symptoms of these strictures or contractions will vary according to the seat and extent of them.

*Case 4. Chronic Bronchitis—Stricture of some of the Bronchia, &c.* A woman, 26 years of age, entered La Charité in the course of September, 1822. She said she had become affected with a catarrhal complaint at the age of 18, which had never left her since. For the first few years it gave her but little inconvenience. At 22 she began to feel dyspnœa, and soon afterwards had a copious hæmorrhage from the lungs. From that period the cough became frequent and distressing—she wasted in flesh and strength, and in the course of the next two years had several pulmonary hæmorrhages. From 24 to 25, Nature seemed to make some stand against the disease, and the symptoms did not gain ground. At 25, she had another severe hæmorrhage, which continued several weeks, more or less. From thence there was rapid loss of strength, and when received into the hospital she was in the last stage of emaciation. By auscultation a cavity was announced under the left clavicle, around which percussion elicited a dull sound. She died three weeks afterwards.

On dissection, vast cavities were found in the *left* lung, around which the parenchymatous structure was hepatised. In the *right* lung, no tubercles existed, and the parenchyma was sound. The bronchia of both lungs were red, and in those of the right lung the following appearances presented themselves. Scarcely had the principal bronchium begun to give off its ramifications, when the parietes of the latter were seen greatly thickened, and their diameters considerably diminished—that is, to one third or one fourth of their natural size. In several places the tubes recovered their proper calibre, and then again became contracted. We pass over our author's attempts to shew how these contractions might have been distinguished in the living body, because they are totally unsatisfactory. But it is interesting to be acquainted with the actual changes themselves, as time and observation afterwards may lead us to appreciate the external phenomena produced by them.

*Case* 5. A man, 31 years of age, entered the hospital on the 31st July 1822, presenting the symptoms of organic disease of the heart. The respiratory murmur was sufficiently audible every where except under the right clavicle, where it was extremely feeble. Percussion was unsatisfactory, on account of the infiltration of the thoracic parietes. The patient said that, for a long time, he had felt a tightness in that side, and an inability to breathe there. In the middle of August, symptoms of hydrothorax came on, with great dyspnœa, and death closed the scene on the 7th September. The right lobe of the lungs was not very crepitous, but otherwise sound. The principal bronchium of this side, a few lines from its origin, was so contracted that the finest probe could scarcely pass through. It then resumed its natural calibre, just before it began to branch off in ramifications. The mucous membrane of the strictured portion was red, and very much thickened. There was nothing else remarkable in the lungs of either side. There was active aneurism (hyperthrophy with dilatation) of both ventricles of the heart —contraction of the origin of the aorta—and general redness of the digestive tube. The sense of tightness in the right side of the chest, and the nearly inaudible state of the respiratory murmur there, were very probably owing to the stricture of the bronchium ; but as these symptoms might be produced by many other morbid states, we cannot set them down as characteristic of the complaint. It is curious that in almost all the instances which have been observed of bronchial contraction, the stricture was in the superior lobe of the *right* lung.

Two other cases are related by our author, in one of which there was *compression* of the large bronchia of the right lung by a mass of encysted melanosis. The respiratory murmur was very feeble in that side. In the other case, there was a total obliteration of some of the bronchia by a similar disease. We shall state the particulars of this last case, on account of the extent of the melanosis.

*Case* 6. A man, 59 years of age, had been affected for many years, with troublesome cough and habitual dyspnœa. He entered La Chrarité in the month of October 1821, in a state of hopelessness. Percussion elicited a very dull sound below the left clavicle, where neither the respiratory murmur nor mucous rattling could be heard. The pulse was small and frequent. M. Lerminier regarded the complaint as pulmonary melanosis. He died in a few weeks.

*Dissection.* The left lung was converted into a black homogeneous mass, of such density that the scalpel would scarcely enter it. The whole appeared like a mass of extravasated black injection after it had cooled, The principal bronchium of this side was sound, as also the three or four first divisions—but further than these no trace of air-tube could be discovered. The rest of the lungs was sound. There are but few cases on record where the melanosis of the lungs went to this extent. We think that M. Lerminier's prognosis, or rather diagnosis was a lucky hit, and not the result of legitimate reasoning or calculation.

4. *Spinal Consumption.* In the sixth Number of the Med. Repos. (New Series) Mr. Gaitskill, of Rotherhithe, has called the attention of the profession to the distinction of spinal consumption from phthisis pulmonalis and tabes mesenterica.

The *cause* of spinal consumption is spinal distortion. Both sexes fall a prey to this lingering and insidious malady—but infinitely more of females than males. In Mr. G's experience the proportion has been nine to one. The years between 14 and 21 are those in which there is greatest danger.

"The foundation of this disease is often laid at the early age of four or five years, and first displays itself in a disposition of the infant to sit or stand with the head inclined forward, but so slightly, as barely to be perceived by the parents. In a few months this inclination becomes noticeable, and the child complains of a little pain in the back, is sick at the stomach, and rather costive, while the muscular flesh wastes. This is attributed to worms, and medical or empirical advice sought for; —if the former be obtained, a few doses of calomel are administered, followed by cooling purgatives and regulated diet. These, with the aid of a recumbent position (which the little patient instinctively covets), afford some temporary benefit." 468.

In this manner the disease will sometimes go on for years, till the stature becomes extended, when the stooping posture becomes conspicuous, when mechanical contrivances, suited well enough for inanimate bodies, are quickly put in requisition. By the aid of generous diet, exercise in the open air, rural amusements, and cold bathing, the progress of the disease is sometimes interrupted, and health ultimately restored. But, on the other hand, it too frequently happens that the muscular power suddenly fails—the bowels become obstinately confined —the animal heat sensibly diminished—the appetite impaired—accession of dry cough—atrophy, and death.

*Diagnosis.* Mr. G. considers that thus the spinal differs from the pulmonary phthisis in many very essential particulars. In the *latter*, there are rigors, succeeded by heat and colliquative sweats, with quick and contracted pulse. In the *former*, there is gradual loss of strength, particularly of the lower extremities—no febrile paroxysms—cool skin (except in the last stage, when the lungs become involved in the disease) —obstinate bowels—slight cough without expectoration; while in common phthisis, the cough is teazing, with muco-purulent expectoration. "It is equally distinguishable, says Mr. Gaitskell, from tabes mesenterica; because in this disease, there is great abdominal fulness, mucous and watery stools, with hectic fever and emaciation; whilst in spinal consumption, instead of abdominal fulness, the bowels are flat and wasted, so that the pulsations of the aorta may be felt through the abdomen."

In respect to the *origin of the disorder*, Mr. Gaitskell has repeated what has now been repeated by many others—forced, as it is, upon the bluntest sense of the most inattentive observer. The rage of the present day to cultivate the mind at the expence of the body, leads to much and irreparable mischief. Such unnatural mental excitation frequently occasions cerebral disorder ending in idiotcy—or, where the chain of connexion is too often lost sight of, in a host of nervous and anomalous complaints, as puzzling to the physician as they are distressing to the patient. The indolence generated by these precocious exertions of the mind, checks the disposition to perpetual motion, so congenial to the young and growing frame, which indolence and its consequences are increased by warm rooms, impure air, and improper diet. Mr. Gaitskell thinks, that another evil is the habit into which children are allowed to get, of reading in a low chair with the book resting on the lap. This leads to a stoop forward, and distortion of the spinal column from its natural and proper shape. Then come the evils of the boarding-school system. Young ladies are packed off to seminaries to allow the mothers time for dissipation at home. At these seminaries they have imposed upon them great restraints, sedentary habits, impure air, scanty diet—while their minds are crammed with ill-assorted knowledge little suited to their years or dispositions. But we must hasten to the *treatment.*

It has been a rule with Mr. Gaitskell, for many years past, when consulted by patients who had lost much flesh by absorption and imperfect chylification, to examine the spine—and, if he found a greater projection than ordinary of the spinal processes, or particular tenderness on pressure with the fingers—or any fixed pain in supporting the body perpendicular to its base—then to suspect "a slow inflammation of the intervertebral substance," which, if not interrupted, leads to alarming evils, such as suppuration, ulceration of the bones, paralysis of the lower extremities, gradual marasmus, and death.

"To stop the progress of this insidious and certainly destructive malady, I have usually recommended the application of four leeches on each side the spine close to the part affected, and when the leeches have fallen off, warm bread and water poultices, till the bleeding ceases. These I have repeated, in the adult subject, every fourth day for a fortnight, till the pain is relieved; and as a deposition of lymph is a natural consequence of every chronic inflammatory action, I have blistered the part two or three times in succession, according to symptoms. These means I have found to be aided by adopting (under certain limitations) the plan of that very excellent surgeon, the late Mr. Baynton, of placing the patient on a horse-hair mattress without a pillow." 471.

As auxiliaries, the warm bath—occasionally an emetic, if the stomach is disposed to reject its contents—a cathartic pill at bed-time (the bowels being confined, as they usually are) purged off next morning with the black draught—after which "it is important to obtain well-regulated hepatic secretions." For this purpose, our author has found five grains of the hydrargyrum cum creta, with an equal quantity of rhubarb every night in a little honey, the best medicine. The action of this is essentially aided by an injection every second night. "It is surprising how much this contributes to promote the proper peristaltic motion of the intestines."

In a month's use of this plan there is generally considerable improvement, and then the patient is allowed to sit up two or three hours daily. As strength increases, walking out of doors is prescribed, with the head upright, or with a weight thereon, as recommended by Mr. Wilson.

We think that, upon the whole, Mr. Gaitskell's advice is judicious.

---

5. *Arachnitis cured.* (Dr. Martinet, Revue Med. Jan. 1824.) Our continental brethren have at length discovered that the symptoms of debility, mental and physical, dependent on inflammation of the sensorium or its coverings, are not to be cured by the "medecine expectante"—nor even by antispasmodics or anodynes—but by blood-letting, local and general—cold to the head—and counter-irritation. This is the "*aujourd' hui*" treatment in France, though it has long been familiar in England. "On ne cherche plus exclusivement, par des antispasmodiques ou autres pretendus calmans, à s'opposer dans ces cas à l'irregularité des phenomènes spasmodiques et au désordre *ataxique* des operations de l'intelligence, qui en constituent les caractères diagnostiques. On attaque directement *aujourd'hui* sa nature inflammatoire, quelle que soit la forme des symptômes sous lesquels elle se presente; et c'est par des evacuations sanguines génerales ou locales, associées la plupart du temps à des derivatifs puissans sur les extrémités, &c. que l'on combat cette maladie." We rejoice to see pathology thus leading to successful practice on the continent, where indeed there is ample scope for improvement in therapeutics. The following cases are not unworthy of perusal.

*Case* 1. Chevillot, aged 48 years, of strong constitution, was taken on the 12th of July, with general malaise, to which was soon added pain in the epigastrium, with vomiting. Twelve leeches were applied to the epigastric region, and the symptoms were removed. 13th. The patient after eating, became affected in nearly the same way. Still there was no head-ache. 14th. He was seized with delirium, and on the 15th he was conducted to the HOTEL DIEU. His symptoms now were, violent delirium, so that several persons were required to hold him. His face was much flushed—great agitation—strong pyrexia—tongue moist. Bled to 32 ounces (this is astonishing in France) and sixteen leeches applied to the neck. In the night, the bandage came off the patient's arm, and two pints or more of blood were lost. 16th. The delirium of a gay kind (delire gai)—constant loquacity—eyes sparkling—pupils natural—agitation general—face rather pallid—pulse full, hard, and frequent. Forty leeches to the neck. Diluents. 17th. The loquacity continues, as also the agitation—tongue and mouth dry—pulse still full and quick. Forty leeches around the temples and forehead. Cold affusion on the head. The delirium now ceased completely, 18th. The countenance calm, and the intellect clear, except the memory, which is imperfect—all the bad symptoms abated—and the straight-waistcoat removed. An erysipelatous inflammation was now discovered on the arm, beginning at the lancet puncture. Twenty-five leeches to the arm—diluents continued—a warm bath. 19th. Convalescent. 20th. Considerable augmentation of the phlegmonous erysipelas on the arm, with appearances of suppuration in different points. Seventy leeches were applied to the arm in the course of the day. Diminution of the inflammation. Incisions were made into the erysipelatous parts in order to give issue to the purulent matter and health was perfectly restored by the end of September.

On the above case we have to remark that, as far as vascular depletion is concerned, the medical officers acted as energetically as on this side of the channel. Local depletion was carried to a greater extent than could well have been practised here, consistent with economy, for the expence of leeches, on such a plan, would be enormous in this country. But it must be obvious that the treatment was defective in respect to purgatives. It does not appear that any aperient medicine was exhibited throughout the whole course of the disease. We think we will be borne out by the experience of practitioners in this country, when we presume that much of the sanguineous depletion might have been saved by active cathartics. A few doses of calomel and colocynth, followed up by salts and senna, would have produced some pints of intestinal secretion, which we verily believe to be equal, on such occasions, to so many pints of blood abstracted from the vascular system.

*Case* 2. A man of strong constitution, a voiturier, 28 years of age, was seized, without any assignable cause, on the 13th July, 1823, with violent pain in the head, which became augmented on the 14th, and accompanied by fever. On the 16th, he was carried to the Hotel Dieu, when the following symptoms were noted. Eyes sparkling and the vessels injected—pupils natural—countenance animated—delirious, so as to require the straight-waistcoat—great loquacity—constant agitation—no convulsions or rigidity of the members—subsultus tendinum —epistaxis—tongue clean, red, and moist—pulse hard and quick—heat of skin moderate—breathing free—chest sounds well. Venesection to 12 ounces (trois palettes) twenty leeches to the neck—cold affusion on the head—whey. The blood was much buffed—the cold affusion was followed by a well marked calm. 17th. Continuation of the delirium, which is of a more lively description—face covered with perspiration, and red—less agitation than yesterday—pulse less rapid. Twenty-four leeches to the temples—the cold affusion. 18th. Quiet delirium—less subsultus—tongue dry, but not red—pulse not much accelerated—heat of skin natural. Twenty-four leeches to the temples—cold affusions. 19th. The same state. 20th. Intellectual faculties restored. 23d. Was convalescent.

The same observations, as to the neglect of purgation, apply to this as to the former case.

*Case* 3. This need not detain us long. The patient was a young girl of seventeen, who, to remove a cutaneous affection from the face, applied the liq. plumb. acet. dil. The eruption being suppressed, she became affected with violent head-ache, delirium, stupor, febrile movements, and diarrhœa. This patient was recovered by only twelve leeches and a blister. *But the diarrhœa continued all the time.*—Another proof how much sanguineous depletion may be abridged by a free state of the bowels.

Dr. Martinet remarks that the diagnostic signs of inflammation of the arachnoid covering the superior portions of the hemispheres, are—disorder, more or less considerable, of the intellect, with irregular movements, preceded, in the great majority of cases, by intense head-ache. The absence of any local loss of sense or motion, on either side of the body, distinguishes this complaint from affection of the cerebral mass itself. Had there existed any inflammation of the thoracic or abdominal organs, the symptoms would have been more complicated, and the arachnitic inflammation might have been entirely masked, and the issue fatal.

---

6. *Physiology and Pathology of the Spleen.* Dr. Vetch, Physician to the Charter-house, has recently published a paper in the Medical and Physical Journal, on the above subject. He concurs in the opinion long ago and still maintained, that the spleen, besides its office in health (whatever that may be) serves as a reservoir for the blood when determined suddenly to the centre from the surface by various accidents, as sudden atmospherical vicissitudes;—or, in certain diseases, as intermittent fevers. But it is to the pathology of this organ that Dr. Vetch's attention is principally directed in the paper before us, as he has had considerable opportunities of observation among our troops, after their return from the fatal swamps of Walcheren. The effects of an enlargement or obstruction of the spleen, Dr. V. thinks, "are such as are most universally referred to the liver." If this be the case, it is doubly necessary to investigate them. But it is to be remembered that, in most of those cases of enlarged spleen, the sequelæ of intermittent fevers, the liver is also affected, either in function or structure, and therefore the practitioner may be led somewhat astray, in keeping his attention strongly fixed on the organ most prominently diseased.

"In enlargement of the spleen, the patient seldom or never complains of much pain in the situation where it might be expected; his appetite is generally good, yet his powers of assimilation are obviously deficient; he loses flesh, and is incapable of any muscular exertion; his features have a peculiar dark, bilious, or mahogany hue, but the conjunctiva preserves its white aud healthy appearance; perspiration is in time wholly suspended, and the skin acquires the appearance and feel of satin;* the lips are pale, and there is generally much wasting of the gums;

---

* "This is observable in convalescents from the remittent fever of warm climates. Vide JACKSON *on Febrile Diseases*."

the urine is limpid, and secreted very rapidly, but contains little or no urea. The patient's mind is generally morose and desponding. All these symptoms of a deranged condition of the spleen, prove that, although the patient may exist for a long time without those changes going on in the blood, which would otherwise take place, the powers of life cease to be renewed. An attack of epistaxis,* or the appearance of moisture upon the skin, are generally signs of returning health. The symptoms I have mentioned are commonly attended with coldness of the lower extremities, generally towards evening, and the pulse is quicker than natural." 443.

A state of the system, such as is above described, Dr. V. has often found in connexion with amenorrhœa—in which cases, an occasional acute pain is liable to occur in the region of the spleen, on any slight exertion. A determination of blood to the spleen is often vicarious of discharges from other parts, in which cases, if long continued, the spleen attains a very large size. In the only three cases where our author saw death take place in the cold state of ague, the spleen was so much distended, and its structure so much altered, that it appeared like a mass of dark uncoagulated blood, which crumbled on the application of the finger. There is a particular form of rheumatism (a modification in fact of intermittent fever) which our author has found to be a source of splenic enlargement. The complaint has been endemic in many places from which ague has disappeared, in consequence of drainage and cultivation. It is a rheumatic affection of the muscular fibres, accompanied by great want of energy in the cutaneous circulation. It seems to have a cold and hot stage, inasmuch as the pain is regularly preceded by a coldness of the parts affected, and also of the lower extremities. It is tedious and untractable, and produces enlargement of the spleen. The most frequent exciting cause of all, in our author's experience, is mental depression and anxiety—long famous, indeed, for causing disorders of the stomach, liver, and spleen—but more frequently, we apprehend, the *consequences* of such disorders.

*Treatment.* Enlargements of the spleen sometimes require evacuations from the neighbourhood, by cupping, leeching, &c. "but a great part of the treatment must consist in obtaining a regular state of the circulation, and at the same time guarding against all causes of excitement until we have removed every symptom of local congestion."

"It is, perhaps, difficult to find any one article of the materia medica, which possesses any great tonic power, which will not, in a short time, require to be discontinued, by its diminishing either alvine discharge, or the secretion of urine. I have, however, been fortunate in finding one very applicable to such a state of disease as I have endeavoured to describe; it is valuable as a tonic, and not less so from the diuretic power, which it never fails to exert, if given in the form to which I confine its use. The article on which I have bestowed this

* "This was very common with convalescents from the Walcheren fever."

panegyric, is a weak infusion of the leaves of the arbutus uva ursi; if the powder of the leaves is substituted, it produces a bitter much too intense to answer the end proposed. I was led to use this medicine extensively, both with officers and soldiers, who had laboured under protracted disease, after the expedition to Walcheren. My subsequent experience has given me ample opportunities of appreciating its value as a medicine, in all cases resembling those I have mentioned. The successful application of small blisters to the epigastric region and left hypochondrium, and the insertion of a seton, are highly useful auxiliaries." 446.

Our author hopes he has now said enough to draw the attention of practitioners to the symptoms attending cases where the system seems to have lost its powers of assimilation. Hysteria, hypochondriasis, mental aberrations, and *diabetes*, are to be found "arising out of primary obstruction of the function of the spleen, as without the co-operation of this viscus, the blood ceases to acquire its recrementitious properties, none of the secretions possess their natural constituent parts, and, what is also worthy of notice, external injuries do not produce the secretion of what is called healthy pus."

We think Dr. Vetch's observations are entitled to much attention; but, considering how intimately linked are the liver, stomach, and spleen, in their pathological ralations, it is possible he may somewhat over-rate the part which the last of these organs plays in the mystic drama of disease.

---

7. *Scirrhus of the Pylorus, and thickening of the Muscular Coat of the Stomach.* [Dr. Louis. Archives, Avril, 1824.] The first case of this kind related by our author, was a woman, forty years of age, who was admitted into La Charité, under Dr. Chomel, on the 18th of September, 1823. She had been ill about twelve months. The complaint began with sense of weight in the epigastrium after eating. This state was soon succeeded by nausea and vomiting—not immediately after dinner, but towards the evening, when most of what she had eaten would come up. The appetite had gradually diminished—emaciation made progress—she felt heat at the pit of the stomach, a disagreeable taste of rotten eggs in her mouth, with nausea and vertigo. Examined at the hospital, she was found greatly emaciated; thirst—anorexia—epigastrium not painful on pressure—to the right, and on a level with the umbilicus, a tumour the size of a man's fist, indolent, and not very sensible to pressure—belly rather more protuberant in the left than in the right side, giving the appearance of the stomach distended—pulse small, feeble, and slow. On the first of October she evinced anxiety; vomiting of bile; pains in epigastrio—flatulence. These symptoms were succeeded by diarrhœa, which disappeared in a few days. On the 21st, she began to vomit, for the first time, a brownish black thick liquid—and next day, there was a great change in the patient's features for the worse. She died two days afterwards.

*Dissection.* The stomach was large, and of an opake whiteness externally. It became suddenly contracted about two and a half inches from the pylorus, and in this contracted and cylindric portion it was very hard, and from 15 to 18 lines in thickness. The stomach contained about two pints of a thick black liquid, similar to that which the patient had vomited, among which were some portions of half-digested aliment. The parietes of the stomach (leaving aside the cylindric portion) were twice their natural thickness, and very firm. This thickness and firmness were seated in the muscular coat of the stomach, throughout its whole extent, but varying in different places. The cylindric portion was so contracted in its calibre, that the point of the little finger could scarcely be introduced. The pylorus was in a state of scirrhus rather than cancer—for ulceration had not actually commenced. The peculiarity of the case consists in the general thickening of the muscular coat of the stomach, examples of which our author could not find in Morgagni, Piort, Chardel, or other authors. He asks, rather pertinently, can this hypertrophy of the muscular coat of the stomach be caused by the exertion of that structure in forcing the digested aliment through a contracted pyloric orifice? Considering how frequent is scirrhous pylorus, and how rare is hypertrophy of the stomach (at least, the notice of such circumstances) we think the theory which would connect them as cause and effect, rather doubtful, notwithstanding that the following case comes in to its support.

*Case* 2. A man, aged forty-one, of small stature, sober habits, thin, and rarely ailing previously, had been, for some tmie, a prey to great anxiety of mind. To this succeeded loss of appetite—uneasiness in the stomach after eating—eructations of an acid kind, especially after taking any wine; but no sense of heat in the epigastrium. When these symptoms had continued about a month, vomiting succeeded meals, at one, two, or three hours' interval. Constipation of the bowels, and great general debility followed. Three months after the commencement of this last train of symptoms, the patient entered the wards of M. Chomel, on the 17th October, 1823. He was now pale, emaciated, highly sensible to the impressions of cold—anorexia—no thirst—pain of the epigastrium, relieved by gazeous eructations, and greatly increased by any distention of the stomach from flatus. A protuberance of the epigastrium, resembling the figure of the stomach, was observable—and an obscure tumour in the region of the umbilicus, cognizable by moderate pressure. The patient frequently ejected a clear fluid from the stomach, and was constipated. After being quiet at the hospital about a fortnight, with proper regimen, and very little medicine, the symptoms were moderated, and the patient could take a little food, being seldom sick afterwards. But this calm was not of long duration. Towards the beginning of December, thirst was complained of—the appetite disappeared—diarrhœa occurred—the epigastric pains augmented—sickness and vomiting increased—and the patient died on the 24th of the same month.

*Dissection.* Stomach voluminous, and descending below the umbilicus, being of a very opake whiteness, and its parietes much thicker and firmer than natural. It became suddenly contracted, as in the former case, about two inches from the pylorus, into a cylindrical form, of great hardness, and from 15 to 18 lines in thickness, admitting with difficulty the point of the little finger. The muscular coat of the stomach was double its ordinary thickness throughout, but without any other appreciable alteration of structure. The pylorus was scirrhous, but not ulcerated.

The two cases above detailed, we grant, are sufficient evidence of such a thing as hypertrophy of the whole muscular coat of the stomach; but other cases will be necessary to prove that the cause is contraction of the pyloric orifice of that organ.

P. S. Since writing the above, we have seen a case published in the *New Bibliotheque Medicale*, by M. Patissier, which bears on the subject in question. A woman, fifty-two years of age, had long laboured under symptoms of scirrhous pylorus, and at length sunk under the disease. On opening the abdomen, M. Patissier was astonished to find the stomach so large as to conceal the whole of the intestines, extending down to the iliac fossæ. When opened, there was a discharge of nearly ten pints of dark-coloured fluid, such as had been vomited by the patient during life. Notwithstanding this extreme distention of the stomach, its parietes were *not attenuated*. The pyloric orifice was scirrhous, and would scarcely admit the point of the little finger. The editor of the journal cites a similar case from Morgagni, and attributes the dilatation of the stomach to the contraction of the pyloric orifice.

